# Training and Technical Assistance Increase the Fidelity of Implementation of a Universal Prevention Initiative in Rural Schools: Results from a 3-Year Cluster-Randomized Trial

**DOI:** 10.1007/s11121-025-01776-0

**Published:** 2025-02-03

**Authors:** Hannah G. Calvert, Christopher M. Fleming, Michaela Lowe, Teri Lewis, Carl F. Siebert, Ashley Havlicak, Nate Anderson, Tate Castleton, Lindsey Turner

**Affiliations:** 1 Institute for Mixed Methods Research, 2110 Artesia Blvd, #191, Redondo Beach, CA 90278 USA; 2 School of Social Work, University of Illinois Urbana-Champaign, Urbana, IL USA; 3 College of Education, Boise State University, Boise, USA; 4 School of Public and Population Health, Boise State University, Boise, USA

**Keywords:** Technical assistance, Virtual learning, Intervention, Implementation, Health

## Abstract

The need for well-implemented evidence-based interventions (EBIs) for the prevention of behavioral issues among children and adolescents is substantial. In rural areas, the need often matches or surpasses that of urban areas. Schools have a wide reach for prevention-focused EBIs. However, implementation in rural schools is often hindered by limited resources and capacity. Rural School Support Strategies (RS3) are a bundle of implementation supports that address implementation challenges in rural settings. They include providing additional leadership and coaching training, individualized technical assistance (mostly virtual), and monthly meetings of a virtual learning collaborative. A cluster-randomized Hybrid Type 3 implementation-effectiveness trial tested RS3 for implementing school-wide positive behavioral interventions and supports (PBIS), a universal prevention approach to improving student behavior, academic outcomes, and school climate. Forty rural schools received a multi-day training on PBIS each summer for 3 years. Half were randomized to also receive RS3 support. Linear and logistic regression models examined the effect of treatment condition and dosage of support on implementation fidelity for Tier 1 (universal) PBIS. Condition and dosage (number of hours) of support increased the odds of schools achieving the 70% threshold for adequate implementation fidelity. In the first year, the higher dosage of technical assistance events increased the likelihood of schools reaching fidelity, whereas later in the trial, the higher dosage of attendance at the virtual learning collaborative sessions yielded significant improvements in fidelity. Implications for accelerating the implementation of universal prevention initiatives in schools—particularly in rural settings—are discussed. This study was prospectively registered on ClinicalTrials.gov (NCT03736395), on November 9, 2018.

**Supplementary Information:**

The online version contains supplementary material available at 10.1007/s11121-025-01776-0.

## Introduction

There is a need for well-implemented evidence-based interventions (EBIs) for the prevention of behavioral issues among children and adolescents. In rural areas, the need often matches or surpasses that in urban areas, given that children and adolescents in rural areas experience similar or greater levels of behavioral health issues including substance use (Lambert et al., 2008), justice system involvement (Blackmon et al., 2016), anxiety, and depression (Figas et al., 2022). Problem behaviors often share common malleable risk and protective factors, and schools are an important setting for establishing safe and nurturing social environments for children and youth. Furthermore, in many rural communities, schools are a central hub for services. Universal prevention programming in schools is important for supporting rural students’ behavioral and mental health needs (Hoover & Mayworm, 2017).

Despite the importance of school-based universal prevention interventions, rural communities face several obstacles to the implementation of EBIs. In rural schools, the implementation of new initiatives is often hindered by limited resources and capacity. In addition to school-level factors, such as leadership and staff capacity, policies and resources at the local and state levels can all contribute to whether scale-up is successful (Fagan et al., 2019). There is expanding interest in leveraging implementation science to enhance the scale-up of EBIs in schools (Bradshaw, 2023; Lyon & Bruns, 2019). Similar to implementation approaches in healthcare and public health (e.g., Durlak & DuPre, 2008; Wandersman et al., 2012), strategies to increase school-level capacity for the implementation of EBIs include both pre-implementation training and other supports during the implementation process, such as technical assistance (TA; Domitrovich et al., 2008; Pas et al., 2023). TA is an individualized approach that builds organizational capacity to implement EBIs (Chinman et al., 2005). Although TA is highly effective for supporting organizations during the implementation of complex interventions (Wandersman et al., 2012), its core features, including timing, dose, and types of implementation strategies used during TA meetings, are still under investigation (Katz & Wandersman, 2016; Le et al., 2016). Learning collaboratives, which allow practitioners to engage (virtually or in-person) with one another as a mutual capacity-building exercise, may also help improve implementation outcomes in school mental health systems (Orenstein et al., 2023; Zubkoff et al., 2019).

### School-Wide Positive Behavioral Interventions and Supports (PBIS)

PBIS is an evidence-based, multi-tiered framework to support students’ well-being and academic learning (Sugai & Horner, 2006). Several randomized trials have shown that PBIS is effective in improving overall school climate (Bradshaw et al., 2008, 2009), as well as the academic and behavioral outcomes of students (Bradshaw et al., 2012; Horner et al., 2009; Noltemeyer et al., 2019; Pas et al., 2019). PBIS promotes safe and effective school environments by establishing a coordinated system for teaching and acknowledging appropriate behavior and establishing predictable consequences for problematic behaviors. As with many other prevention initiatives (Domitrovich et al., 2008; Hoagwood & Johnson, 2003), PBIS uses a three-tiered continuum of support, consistent with a public health and prevention science approach, to reduce risk factors and increase protective factors to prevent the progression of problematic behaviors and academic underperformance. At Tier 1, efforts involve supporting all students and changing the whole school environment. Advanced tiers (Tiers 2 and 3) provide targeted and intensive interventions for students who need additional support (Sugai & Horner, 2006).

PBIS is disproportionally adopted in urban and suburban schools compared to rural schools. Rural schools represent about 20% of schools implementing PBIS (Chaparro et al., 2022) despite making up 32% of all public schools (National Center for Education Statistics, 2022). Geographic distance and funding constraints pose challenges for access to professional development and TA. However, rural communities also have unique strengths that make universal programming effective, including smaller schools with lower student-to-staff ratios (Molloy et al., 2013), close-knit communities, innovative problem solving, and community pride (McDaniel & Bloomfield, 2020b). Implementation science methods and frameworks can answer questions about how to improve the scale-up of EBIs in schools, particularly in rural areas.

### Study Purpose

Few RCTs have been conducted investigating strategies for improving the implementation of universal EBIs in schools (Baffsky et al., 2023a, 2023b). The few that have been conducted show that training combined with consultation and coaching (i.e., TA) are effective strategies for improving implementation fidelity (Becker et al., 2014; Bradshaw et al., 2008, 2010) and similar results have been found in non-randomized trials (McDaniel & Bloomfield, 2020a; von der Embse et al., 2019). However, the literature on the relative effectiveness and dosage of different types of capacity-building strategies for EBIs in schools is underdeveloped, particularly for rural schools (Cook et al., 2019; Domitrovich et al., 2015). The purpose of the current Hybrid Type 3 trial was to examine the relative effectiveness of a bundle of implementation supports called Rural School Support Strategies (RS3; Turner et al., 2022) on school-level implementation of a universal prevention initiative in rural schools, over 3 years. The primary outcome was the fidelity of PBIS implementation at Tier 1. Analyses explored the following research questions: (1) To what extent do the implementation supports, overall and individually by type, improve schools’ implementation fidelity outcomes each year? (2) Do the implementation supports, overall or individually by type, make schools more likely to achieve 70% implementation fidelity? We hypothesized that—relative to schools receiving training only—the schools receiving RS3 supports would show (1) significantly greater increases in fidelity and (2) would be more likely to reach the 70% threshold considered sufficient for fidelity. Exploratory analyses examined how the dosage of RS3 components affected fidelity.

## Methods

The trial took place during the 2019–2020, 2020–2021, and 2021–2022 school years. All 40 participating schools received basic support for PBIS implementation, which included guidance in assembling a building-level implementation team (five to eight school staff, including an administrator) and yearly team trainings on the PBIS framework. The 20 schools randomized to the intervention received the RS3 supports in addition to training (described below). The trial tested a “blended strategy” (Proctor et al., 2013) relative to active “treatment as usual.” We describe the arms of the trial as “basic support” or “RS3” rather than as control and intervention.

Hybrid trial designs are used to rigorously examine interventions to improve the implementation of EBIs (Curran et al., 2012). A cluster-randomized Hybrid Type 3 design primarily examines strategies and implementation outcomes at the organization level while also testing individual-level outcomes relevant to the EBI being implemented. The original trial was designed to examine student-level outcomes including behavior, perceived climate, and academic outcomes (i.e., individual-level measures of the clinical effectiveness of the EBI); however, due to pandemic-related disruptions to education systems, these measures could not be collected during the trial. Consequently, analyses examined only organizational-level implementation outcomes (i.e., fidelity). The study protocol is described in detail elsewhere (Turner et al., 2022), with a description of pandemic-related impacts using the CONSERVE (CONSORT and SPIRIT Extension for RCTs Revised in Extenuating Circumstances) guidelines (Orkin et al., 2021). The trial was intended to occur over 2 years, but due to the pandemic, a funding extension was granted, allowing the intervention to continue for a third year. Two of the 40 schools declined to continue for the optional extension.

### Theoretical Framework

This study of RS3 was based on the Interactive Systems Framework for Dissemination and Implementation (ISF; Wandersman et al., 2008) which specifies three systems that interact to support the implementation of evidence-based prevention practice. The research group served as (1) the synthesis and translation system (distilling evidence into actionable strategies for the deliverers) and (2) the prevention support system (providing deliverers with technical assistance throughout implementation), and schools served as (3) the delivery system. The Quality Implementation Framework (Meyers et al., 2012) details actions to be taken by each of the three parts of the ISF system during four phases of implementation: (1) assess context and build capacity; (2) create a structure for implementation, such as teams; (3) provide ongoing structure as implementation occurs; and (4) improve future applications. We used both frameworks to conceptualize the design of the trial, the RS3 intervention, the process measures, and the philosophy to guide how our team interacted with educators (i.e., the delivery systems).

### Basic Supports and Rural School Support Strategies (RS3)

#### Teaming

All schools were guided in developing a PBIS implementation team of five to eight people, including the school principal and one coach who worked together to guide the team.

#### Yearly PBIS Trainings

All schools were provided trainings on PBIS content in the summers of 2019, 2020, and 2021. The content was aligned with PBIS Tiers, with Year 1 covering Tier 1 (universal prevention), Year 2 covering Tier 2 (targeted interventions) plus a refresher on Tier 1, and Year 3 covering Tier 3 (intensive) interventions with a Tier 1 refresher and integration of practices across all tiers. Training was conducted in-person in 2019 and virtually in 2020 and 2021. All members of each school’s implementation team attended the trainings. Schools were grouped by treatment condition and geographic region. Activities included didactic lectures, full-group problem solving, and time for teams to collaborate and build their action plan. Trainings lasted 3–4 days and occurred across seven different locations (for in-person trainings in year 1; and regional groupings were maintained for the subsequent virtual trainings) over the course of seven weeks during June and July.

#### Technical Assistance (TA)

Proactive in-person and remote TA was delivered on a monthly basis to RS3 schools by two implementation support practitioners (ISPs; Albers et al., 2020; Metz et al., 2021). The ISPs were K-12 educators with strong content expertise and credibility, having previously worked in rural Idaho schools leading PBIS implementation. During the first 3 months of the trial, ISPs traveled to the RS3 schools for in-person TA visits. In the winter of 2019, visits transitioned to a virtual format (Zoom) due to the hazards of traveling to remote mountainous regions in winter. The trial design planned a mix of TA provided primarily through a virtual modality with occasional onsite TA visits. However, the COVID-19 pandemic forced all TA to be delivered virtually after the first 3 months. In the third year of the trial, supplemental TA was provided by an additional ISP with expertise in advanced tiers of PBIS (targeted and intensive interventions for selected students).

TA was tailored to each school’s needs, generally including guidance on data-based decision-making, problem solving, following their action plan, and engaging in audit and feedback. Typically, each school’s assigned ISP would attend the team’s monthly meeting (in-person at first, then virtually), and coaches could email or call their ISP. Although ISPs proactively contacted RS3 coaches every month, each school varied in the amount of TA used.

#### Virtual Learning Collaborative (VLC)

The ISPs hosted monthly meetings of a virtual learning collaborative (VLC; Zubkoff et al., 2019) for RS3 coaches. Each hour-long session began with a presentation on a specific topic (e.g., coaching strategies, PBIS refreshers, wellness). In the second half hour, coaches were given time to share experiences, solve problems, and ask the ISPs questions. Attendance averaged 12 coaches per meeting.

#### Additional Year 1 Didactic Trainings

The coach and administrator at RS3 schools received three additional in-person trainings in the first year. The first focused on planning for implementation. The second two focused on coaching school teams through implementation. Each training lasted 1 full day and was attended by all RS3 schools. As there was no variability in attendance, this element of RS3 was not included in dosage analyses.

#### Online Resources

RS3 schools had access to a password-protected web portal with resources (e.g., videos, forms). Log-in data showed that utilization was low, mainly occurring after the ISPs had referred coaches to resources during TA meetings, thus dosage was not included in analyses as it was already reflected by the TA calculations.

### Sample

**Fig. 1:**
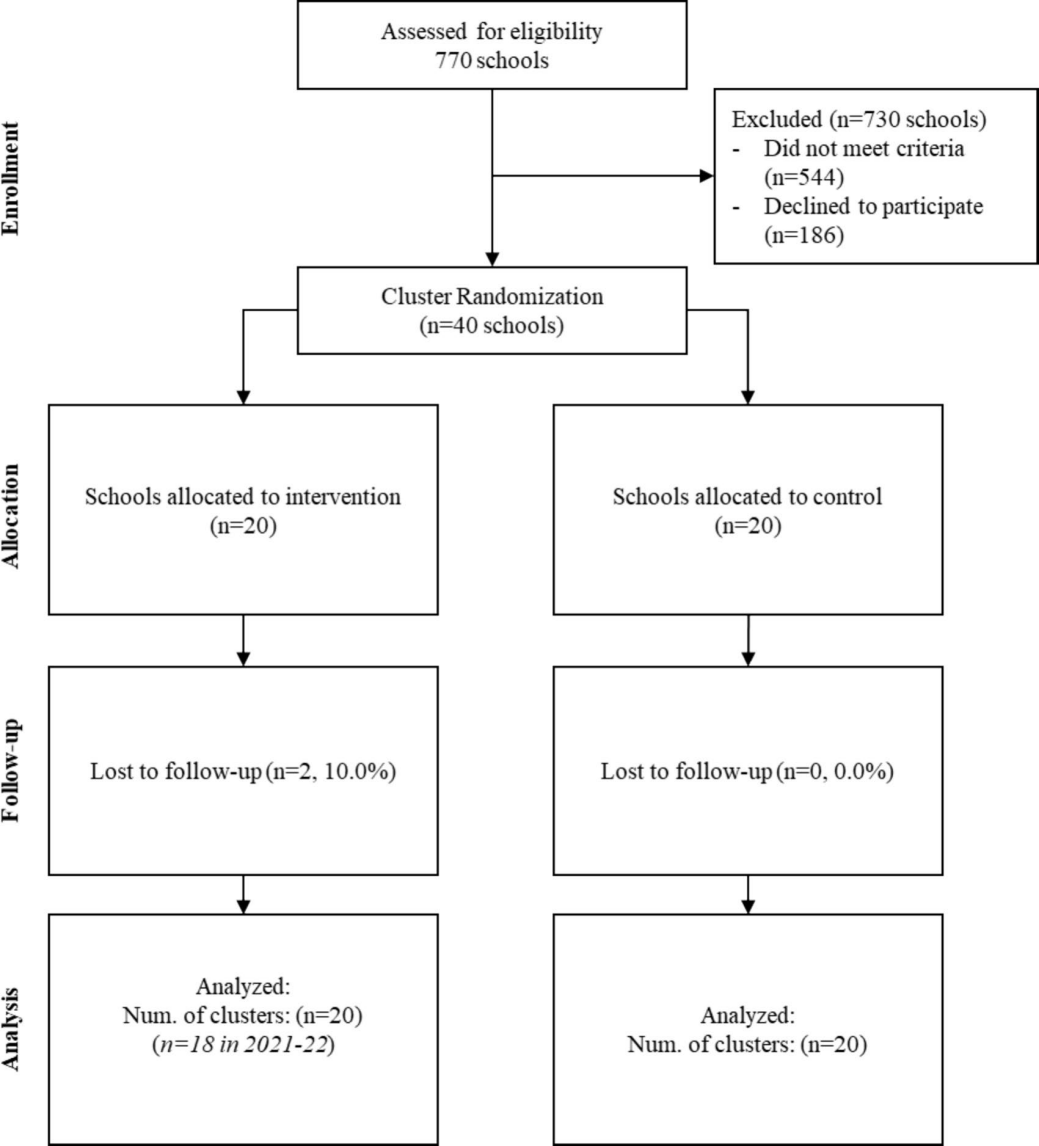
CONSORT diagram

Demographic characteristics of all rural, public K-12 schools in Idaho were obtained from the Common Core of Data (National Center for Education Statistics, 2021). In the fall of 2018, all public schools serving any grade level from Kindergarten to Grade 12 were invited to apply for the trial if they were located in a rural area or township, had at least 100 students, and had no prior PBIS training. Invitations were sent via mail and email and were followed up by phone calls. Forty schools were recruited. This sample size was planned to allow sufficient power (> 0.80) to detect a small-to-medium (*d* = 0.30) effect size in a school-level regression model. Once 40 schools were recruited (three additional schools were waitlisted) and each school’s principal and district administrator completed a memorandum of understanding and consent to participate, the schools were pair-matched by size (number of students), poverty level (percent of students eligible for free or reduced-price lunches), and grades served. Pair-matching also accounted for district membership. One school (or district, for district-level blocking) within each pair was randomized to receive RS3 and the other to basic support, via coin flip. The randomization procedure was conducted by project staff and observed by three independent researchers including a doctoral-level statistician. Chi-square and *t*-tests confirmed that demographic characteristics (number of teachers, number of students, and percentage of students eligible for free/reduced-price meals) were not significantly different between groups. Generally, schools were small, with just under 350 students and 19 teachers, on average. Most were rural remote (42.5%; more than 25 miles from a metropolitan area) or rural distant (40%; 5 to 25 miles from a metropolitan area). Most were K-5 or K-8 schools (57.5%), with 20% middle schools, 10% high schools, and 12.5% serving all grades K-12. The CONSORT diagram is shown in Fig. 1.

Consistent with the racial demographics of rural Idaho, schools served predominantly White student populations (range = 33–97%; *M* = 77%, SD = 16%), with substantial Hispanic (*M* = 19%, SD = 16%) representation, and other races reported at ≤ 2% on average. Socioeconomic characteristics of the communities served by each school varied, as proxied by the percentage of students eligible for free/reduced-priced school meals. To maintain school anonymity, this indicator is reported by collapsing into three groups: 35% of schools had < 40% eligibility (lower poverty), 42.5% had 40–59% eligibility, and 22.5% had ≥ 60% eligibility (higher poverty).

### Measures

#### Dosage of Implementation Support

Attendance at trainings, TA events, and VLC sessions was documented by the project team. Trainings and VLC sessions had a set duration for each occurrence (8 h per training and 1 h per VLC session). Individual TA meetings were variable. Each TA event was documented by the ISPs using an online tracking log including the type of meeting (in-person, zoom, or phone), recipient(s), duration, and notes on the content and topic of the meeting. Five VLC sessions were held in 2019–2020, and nine occurred each year thereafter (VLC sessions were not offered monthly as intended in spring 2020 due to pandemic-related disruptions). The dosage of support was calculated for each school, conceptualized as the number of hours of implementation support, both within type and total. Table 1 shows descriptive statistics for implementation support events for RS3 schools.

**Table 1:** Dosage (number of hours) of implementation supports; RS3 schools only (*n* = 20)

Year and type	Dosage (number of hours)			
	Min	Max	Mean	SD
**2019–2020**				
Additional trainings	24.0	24.0	24.0	0.0
VLC sessions	0.0	5.0	3.7	1.6
TA events (total)	4.0	34.0	10.1	7.0
*In-person*	2.5	28.0	7.2	6.2
*Virtual*	1.0	6.0	2.7	1.3
*Phone*	0.0	3.0	0.3	0.7
Total	30.0	63.0	37.8	7.7
**2020–2021**				
VLC sessions	0.0	9.0	6.1	3.3
TA events (total)	0.0	5.5	3.5	1.5
*In-person*	0.0	0.0	0.0	0.0
*Virtual*	0.0	5.5	3.3	1.5
*Phone*	0.0	1.8	0.2	0.5
Total	0.0	14.3	9.5	4.2
**2021–2022**				
VLC sessions	0.0	9.0	4.2	3.0
TA events (total)	1.0	8.5	4.3	2.0
*In-person*	0.0	1.0	0.1	0.3
*Virtual*	1.0	8.0	4.1	1.9
*Phone*	0.0	0.7	0.1	0.2
Total	2.0	14.8	8.5	3.5

*Note. VLC* virtual learning collaborative, *TA* technical assistance

#### Implementation Fidelity

Fidelity was assessed with the Tiered Fidelity Inventory (TFI; Algozzine et al., 2019). The TFI was designed for coaches and administrators to assess the degree to which they are adopting and applying core components of PBIS. For this study, school teams completed the TFI each year during summer training institutes. The TFI has high content validity, inter-rater (ICC = 0.99) and test–retest (ICC = 0.99) reliabilities (McIntosh et al., 2017), and convergent validity with other Tier 1 PBIS evaluation tools (*r*s from 0.65 to 0.96; Mercer et al., 2017). Because Tier 1 practices have the most robust associations with improved behavioral outcomes (Lee & Gage, 2020), the present analyses focus only on Tier 1 implementation fidelity. Tier 1 of the TFI includes 15 items across three subscales (teams, implementation, evaluation), and recent literature suggests that considering fidelity at the subscale level within each tier may provide the most accurate representation of fidelity (Grasley-Boy et al., 2023). Activities measured by the teams subscale and the evaluation subscale were largely facilitated by the trial (i.e., expected for schools in both conditions), so we present data from the 9-item Tier 1 implementation subscale of the TFI as a standalone outcome. This subscale assesses the degree to which the school is instituting the core components of PBIS, including defining problem behaviors and expectations, and establishing a system for feedback. Each item is rated 2, 1, or 0 points for fully, partially, or not implemented. We examined the total Tier 1 TFI score (hereafter: TFI) and implementation subscale in two ways: as a percentage (0–100%) of total possible points and as a dichotomous variable indicating whether the school met or surpassed the 70% threshold, which was suggested by the tool’s creators (Algozzine et al., 2019) and is associated with improved student outcomes (Grasley-Boy et al., 2022; Noltemeyer et al., 2019).

#### School Characteristics

Since implementation fidelity is typically higher in primary schools (i.e., Grades K-6) (Molloy et al., 2013), we included school level in the models as a binary covariate (K-5/K-8 schools = 1; middle/high/K-12 schools = 0).

### Analysis

We first examined school-level fidelity using the total TFI and implementation subscale in each year, for basic support schools and for RS3 schools, using *t*-tests to assess whether fidelity differed significantly within-year, unadjusted, and by condition. We also examined the number of schools meeting the 70% threshold at each time point. Thereafter, descriptive statistics were used to explore the dosage of implementation support events, considering how much variability existed in the amount of exposure to the types of support each year, among RS3 schools only. To assess the impact of RS3 on fidelity, we calculated a series of multivariable regression models to explore changes in fidelity in relation to exposure to varying dosages of support during that school year. First, we used sequential linear regressions to assess the overall condition effect, testing whether the RS3 condition improved scores on the TFI and implementation subscale each year, controlling for school level and the prior year’s score on the same scale. Next, linear regressions tested whether the dosage (duration of training, TA, and VLC events attended each year) predicted improvement, including the same controls. The calculation of total dosage in 2019–2020 included three trainings (adding a constant across all RS3 schools) in addition to variable dosage of TA and VLC events for each RS3 school. In subsequent years, dosage included only TA and VLC attendance because supplemental trainings were not offered. Model fit was assessed with *R*^2^ values and *F*-tests.

Lastly, to test whether each school attained sufficient fidelity of implementation, we re-calculated each model with logistic regression, using the 70% threshold to create a binary outcome (≥ 70% = adequate fidelity). Model fit was assessed with pseudo-*R*^2^ (Cox & Snell) values and omnibus chi-squared tests. Analyses were conducted on a year-to-year basis rather than examining trends over time with longitudinal modeling approaches, such as growth curve modeling. This was a modification of our pre-registered protocol (Turner et al., 2022) and was deemed necessary due to the non-linear pattern in TFI scores over time, due to the pandemic.

## Results

**Fig. 2:**
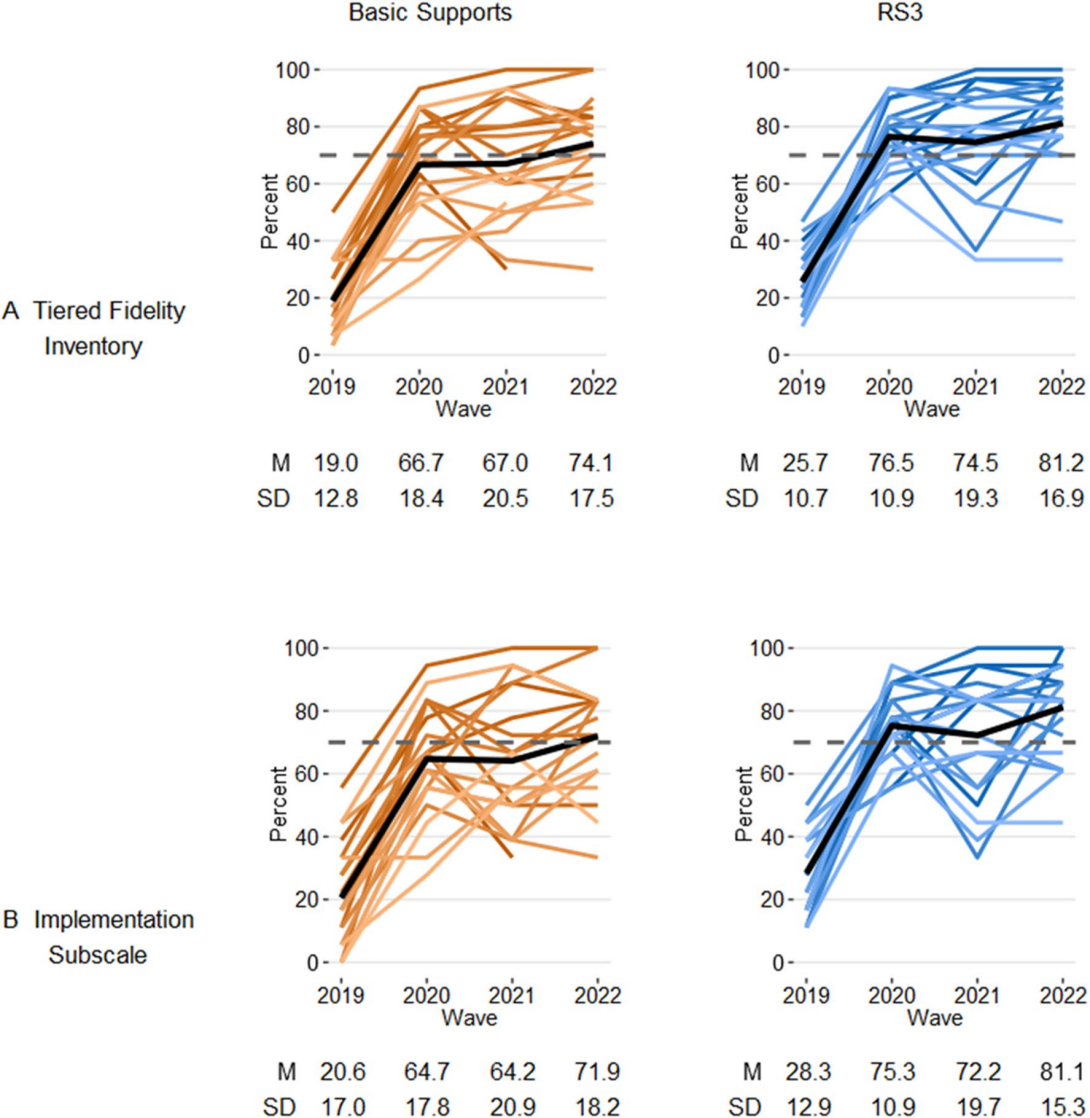
Fidelity outcomes: percentage of total points, by scale and condition. *Notes*. Lines portray individual schools. Black lines show the scale mean value within the condition. Dashed gray line indicates a 70% threshold

Figure 2 shows TFI and implementation subscale scores over time at all 40 schools in the study, separated by treatment condition. At baseline, RS3 schools had slightly greater TFI scores than basic support schools (*t*(38) = 1.79, *p* = 0.08), but none met the 70% threshold. At the end of the first year, scores were greater for both RS3 and basic support schools and were significantly greater at RS3 schools for the TFI (*t*(30.8) = 2.05, *p* < 0.05) and implementation subscale (*t*(38) = 2.30, *p* < 0.05) compared to basic support schools. At the end of the first year, 16 RS3 schools met the 70% threshold for total TFI, compared to 11 basic support schools (*χ*^2^(1) = 2.85, *p* = 0.09), and 15 RS3 schools met the 70% threshold for implementation, compared to seven basic support schools (*χ*^2^(1) = 6.47, *p* = 0.01). TFI and implementation scores declined slightly in 2021, before increasing slightly for both conditions in the final year. In 2021, fewer schools in RS3 and basic support met the 70% threshold for TFI (14 and 9, respectively) and implementation (12 and 7, respectively) than the prior year, but by 2022, most schools met this threshold for TFI (18 and 13) and implementation (15 and 11). Condition differences were not significant in either year.

### Linear Regression Models to Predict Implementation Percentage Scores

#### Effect of RS3 Condition

Table 2 shows the main effects from a series of linear regression models testing the effects of treatment condition— and dosage of implementation support elements—on the TFI total and implementation subscale scores. The RS3 condition had a marginally significant effect on both the TFI and implementation subscale in 2020 only, corresponding with a TFI percentage score that was 8.48 points higher among RS3 schools than basic support schools and an implementation subscale score that was 8.54 points higher than basic support schools in the first year of the trial.

**Table 2:** Summary of results of separate linear regression models including key predictor variables on the Tiered Fidelity Inventory Tier 1 total score and Tiered Fidelity Inventory Tier 1 implementation subscale score

	Tiered Fidelity Inventory, total score					Implementation subscale, subscale score				
	Coefficients for key predictor in each model				Model fit	Coefficients for key predictor in each model				Model fit
	*β*	*b*	SE	*p*	*R*^2^	*β*	*b*	SE	*p*	*R*^2^
**2019–2020**										
RS3 condition	**.27†**	8.48	4.61	.074	.27	**.28†**	8.54	4.45	.063	.29
Total dosage	.19	0.15	0.12	.212	.24	.25	0.19	0.12	.107	.27
Dosage of VLC	.12	0.88	1.12	.436	.22	.17	1.23	1.07	.259	.25
Dosage of TA	.03	0.07	0.35	.845	.21	.15	0.32	0.34	.342	.24
**2020–2021**										
RS3 condition	.01	0.41	5.57	.942	.36	.09	3.74	6.42	.564	.21
Total dosage	− .05	− 0.18	0.49	.723	.37	− .02	− 0.07	0.57	.907	.20
Dosage of VLC	− .06	− 0.31	0.73	.670	.37	− .03	− 0.18	0.84	.832	.20
Dosage of TA	− .03	− 0.25	1.35	.854	.36	.01	0.11	1.56	.943	.20
**2021–2022**										
RS3 condition	.12	4.06	3.81	.294	.58	.19	6.38	4.39	.155	.44
Total dosage	.13	0.45	0.39	.252	.59	.18	0.63	0.45	.167	.44
Dosage of VLC	**.21†**	1.22	0.61	.055	.62	**.25†**	1.42	0.71	.053	.47
Dosage of TA	.00	− 0.01	0.75	.992	.57	.05	0.35	0.87	.690	.41

*Notes*. †*p *< .10 *p < .05 **p < .01 ***p < .001. Model fit (*F*-test with 3*df*) was significant at *p *< .05 or better for all models. Each model controls for two covariates: school level (K-5/K-8=1); and prior year’s TFI score. *TA* Technical Assistance, *VLC* Virtual Learning Collaborative

#### Dosage: Number of Hours of Implementation Support

No significant effects were observed for the total dosage of implementation supports on either the TFI or implementation subscale. However, marginally significant effects were observed when examining the dosage of VLC sessions in 2021–2022, for both outcomes. VLC dosage models are interpreted as each hour of VLC attendance being marginally associated with increasing TFI and implementation scores by 1.22 and 1.42 points, respectively.

### Logistic Regression Models to Predict Sufficient Implementation (70% Threshold)

Table 3 shows models exploring whether intervention components were associated with the likelihood of schools reaching the 70% threshold for fidelity of implementation. Neither the overall intervention nor the dosage of any particular components was associated with an increased likelihood of schools attaining the 70% threshold on the TFI. Several intervention components were associated with attaining 70% on the implementation subscale in 2020. There was a significant effect for the overall RS3 intervention (AOR = 6.23, *p* < 0.05), while VLC dosage was marginally significant (AOR = 1.34, *p* = 0.098). There was a significant effect for the overall dosage of implementation supports (AOR = 1.05, *p* < 0.05) and marginal effects for the dosage of TA (AOR = 1.19, *p* = 0.056). There were no significant effects in subsequent years.

**Table 3:** Summary of results of separate logistic regression models including key predictor variables on Tiered Fidelity Inventory threshold of 70% fidelity

	Tiered Fidelity Inventory, 70% threshold					Implementation subscale, 70% threshold				
	Coefficients for key predictor in each model			Overall model fit		Coefficients for key predictor in each model			Overall model fit	
	AOR	95% CI	*p*	*R*^2^	*χ*^2^	AOR	95% CI	*p*	*R*^2^	*χ*^2^
**2019–2020**										
RS3 condition	3.42	0.70, 16.67	.128	.20	8.70*	6.23*	1.32, 29.43	.021	.26	11.90**
Total dosage	1.02	0.98, 1.06	.333	.17	7.21†	1.05*	1.01, 1.09	.025	.25	11.67**
Dosage of VLC	1.09	0.76, 1.55	.654	.15	6.45†	**1.34†**	0.95, 1.89	.098	.20	8.78*
Dosage of TA	0.99	0.88, 1.11	.852	.15	6.28†	**1.19†**	1.00, 1.42	.056	.24	10.75*
**2020–2021**										
RS3 condition	2.05	0.42, 10.02	.374	.32	15.57**	2.11	0.45, 9.90	.345	.30	14.00**
Total duration	1.00	0.87, 1.15	.965	.31	14.77**	0.98	0.85, 1.12	.724	.28	13.23**
Dosage of VLC	0.96	0.78, 1.18	.703	.31	14.91**	0.93	0.76, 1.15	.512	.29	13.54**
Dosage of TA	1.12	0.76, 1.65	.557	.32	15.12**	1.05	0.72, 1.53	.797	.28	13.17**
**2021–2022**										
RS3 condition	4.26	0.36, 50.86	.252	.36	16.93***	1.73	0.25, 12.24	.581	.43	21.28***
Total dosage	1.18	0.89, 1.58	.257	.36	17.03***	1.06	0.87, 1.28	.588	.43	21.27***
Dosage of VLC	1.64	0.69, 3.90	.261	.38	18.12***	1.30	0.88, 1.92	.184	.45	22.97***
Dosage of TA	1.15	0.73, 1.81	.534	.34	15.86**	0.93	0.66, 1.31	.677	.43	21.14***

*Notes.* Each model controls for two covariates: school level (K-5/K-8 = 1); and prior year’s TFI score. *R*^2^ values reflect Cox & Snell pseudo-*R*^2^; χ^2^ values reflect omnibus tests with 3*df*

*AOR* adjusted odds ratio, *CI* confidence interval, *TA* technical assistance, *VLC* virtual learning collaborative

^†^*p* < .10, *p < .05, **p < .01, ***p < .001

## Discussion

We found that basic implementation supports—establishing an implementation team and hosting intensive, team-based training—helped rural schools build a strong foundation for the implementation of an evidence-based universal prevention initiative. However, additional supports significantly accelerated implementation fidelity. Consistent with prior literature (Becker et al., 2014; Bradshaw et al., 2008, 2010), as well as best practice guidelines for implementing PBIS (Fixsen et al., 2005) and other EBIs, we found that initial training promoted increases in implementation fidelity, but schools that received a comprehensive set of supports had significantly higher fidelity in the first year and maintained higher fidelity in years 2 and 3 (although not statistically significant). As one of the few studies to systematically measure and examine the dosage of implementation supports provided over time, our results, while necessarily interpreted with the caveat of the pandemic, provide important insights for optimizing the timing and quantity of supports to help schools to implement school-wide EBIs.

The TA provided in our study was proactive, customized, collaborative, and long-term, all of which are aspects noted by Wandersman and colleagues (2012) as important for TA effectiveness. Our results showed that TA had a significant effect on schools reaching 70% fidelity in year 1, whereby schools that utilized more TA were more likely to attain sufficient fidelity. School teams used more TA in year 1 than in years 2 and 3 (10.1, 3.5, and 4.3 h on average, respectively), while the range between minimum and maximum hours of TA use by year was 30, 5.5, and 7.5 h. This underscores the importance of providing flexible TA based on need, with a focus on intensive support during early implementation, as suggested in TA models (e.g., Le et al., 2016). The fact that there was not a continued significant effect of the TA in later years is also supported by growing evidence that associations between use of supports and team/coalition functioning are not always linear throughout implementation phases (Olson et al., 2020). Chilenski and colleagues (2021) recently showed that a higher dosage of TA (measured as the frequency of contact) early on was related to worse team functioning in the early stages, but that same high dosage of early TA was related to better team functioning later in the implementation. It is likely that TA utilization (i.e., dosage) in our study was related to implementation fidelity in the first year because schools were only focused on Tier 1 during the first year, and no schools had achieved 70% implementation fidelity yet. In subsequent years, it becomes harder to interpret the relationship between utilization of TA and fidelity of Tier 1 implementation, because it is likely that some schools continued to implement well without needing much TA, whereas others struggled while using comparatively more TA. The qualitative evidence from our trial shows that school coaches and administrators found TA to be the most acceptable and appropriate form of implementation support (Calvert et al., 2023).

The research examining the effectiveness of virtual learning collaboratives as a support for improving fidelity of universal prevention EBIs in schools is growing, but still relatively nascent (Baffsky et al., 2023a). Our approach for conducting VLC sessions included elements of training (in the form of revisiting/refreshing content) and TA, while also providing an outlet for other implementation strategies. School coaches could share data, receive feedback, and problem-solve with ISPs as well as coaches from other RS3 schools. Our results showed that higher VLC attendance predicted higher odds of reaching 70% implementation fidelity in the first year, and attendance had a marginally significant linear relationship with fidelity in year three. Average rates of attendance were similar in all study years (3.7, 3.5, and 4.3 h, respectively). The fact that the VLC was the only implementation support with a relationship (albeit marginally significant) with implementation fidelity in the final year of the trial shows the strength of these scheduled and predictable virtual communities for supporting sustained fidelity years after initial implementation. Studies have shown VLCs to be feasible and effective for supporting implementation, although the discrete implementation strategies that VLCs employ can make direct comparisons of effectiveness challenging (Connors et al., 2020; Orenstein et al., 2023). Qualitative data from the trial showed that the acceptability of VLC sessions was somewhat lower than for TA; however, the VLC was received more favorably over time, due to attendees developing deeper relationships with their peers from other RS3 schools (Calvert et al., 2023). The potential higher acceptability and feasibility of our VLC compared to other types of peer learning groups for school personnel (e.g., Baffsky et al., 2023b) might be due to the strategies used; our VLC was proactively organized and led by an ISP, so coaches only had to virtually “show up” to have a tailored learning opportunity and protected time to interact with their peers.

Our findings on the effectiveness of the RS3 implementation supports align with other research on implementation strategies in school settings. A comprehensive taxonomy of implementation strategies was identified in the Expert Recommendations for Implementing Change (ERIC) project (Powell et al., 2015) and then further refined for school-specific applications in the SISTER (School Implementation Strategies, Translating ERIC Resources) taxonomy by Cook and colleagues (2019). A recent systematic review of empirical evidence on the effectiveness of strategies for implementation of school-based mental health EBIs identified 21 articles (five randomized trials), with most using SISTER strategies of *conduct ongoing training* and *provide ongoing consultation/coaching* (Baffsky et al., 2023a, 2023b). Based on the results of those studies, Baffsky and colleagues identified several promising strategies, including strategies used in our project such as engaging in audit and feedback (done by our TA providers) and engaging a school leadership team. They did not find any published evidence about the effectiveness of *create a professional learning collaborative*. While the TA and VLC sessions were a medium for engaging in a variety of strategies (i.e., educating, coaching, auditing, organizing; see Turner et al., 2022 for a full list of identified SISTER strategies), due to the breadth of strategies used and long timespan of the study, we were unable to collect data regarding the effectiveness of all discrete strategies used. This highlights the need for further research on the types of activities that occur during TA and VLC sessions, and how they align with SISTER strategies. Future mixed-methods studies could improve knowledge about which strategies actually work, through protocolized approaches testing the use of discrete implementation strategies (e.g., audit/feedback versus information only) during TA visits, rather than our blended approach.

### Strengths and Limitations

This trial was developed in line with the standards of evidence of the Society for Prevention Research (Gottfredson et al., 2015), emphasizing rigor in the design, which included cluster-randomization, longitudinal data collection, careful documentation of intervention components (i.e., implementation supports offered and delivered), the use of an active control condition, a focus on understudied rural schools, and baseline data collection that included measures of community context. However, there are several limitations, many of which stem from pandemic-related impacts. Pertaining to the outcome of fidelity, we designed the trial with a plan to annually collect two measures of fidelity, both created by the PBIS developers and widely used in the field. In addition to the staff-reported TFI, we planned to use the Schoolwide Evaluation Tool (SET; Sugai et al., 2001) to assess fidelity. The SET is completed by trained observers who are masked to treatment condition, which would have allowed an independent and unbiased outcome measure. Although SET data were collected from all schools at baseline, pandemic restrictions made on-site follow-ups impossible.

As previously noted, the planned activities of the trial—including student-level data collection—were altered due to the pandemic. While the sample size of 40 schools is substantial for an implementation study, it is insufficiently powered for complex analyses involving only organizational-level outcomes. Our study was designed to test how changes in PBIS fidelity impacted student outcomes, but student-level outcomes were not available. Recent modeling simulations (Williams et al., 2022) confirm that at least 40 cluster-level cases (i.e., schools) and 600 individual-level cases (i.e., students) are needed in three-level modeling to assess mediational mechanisms of change. Those simulations suggest that our original design would have been adequately powered; however, while the sample size of 40 schools is substantial for an implementation study, it is insufficiently powered for complex analyses involving only organizational-level outcomes. Further, we were not able to adjust for family-wise error inflation. Although the random allocation to condition resulted in similar demographics across condition, other covariates—including ones that we measured such as community context and resources—may have accounted for observed variation in outcomes, but we could not add more covariates without overparameterizing the models. Because this study focused on the scale-up of a universal prevention intervention in rural schools in Idaho (small schools with a majority of White students and moderate socioeconomic disadvantage), these findings may not generalize to all rural areas. It is also important to note that fidelity is an important implementation outcome for EBIs, but without adaptability, EBIs may not be effective, appropriate, or a good fit in various settings. This fidelity-adaptation dilemma is well-known in prevention science (Dane & Schneider, 1998) and is also an essential consideration in implementation research, particularly in experimental trials where fidelity is a key outcome. In the current study, the EBI itself is inherently adaptable, given that PBIS is a framework, and fidelity is measured by the presence of “key features” (i.e., core components). Our interventionists encouraged adaptation based on each school’s context and need but such an approach may not be appropriate with other EBIs.

## Conclusions

Intensive team-based trainings facilitate the implementation of EBIs in rural schools, but fidelity is accelerated by additional supports, including TA and learning collaboratives. We found that the impacts of TA and VLCs on fidelity were significant even though most supports were provided virtually in the first year, which shows the value of providing hybrid online and in-person support for schools implementing an EBI. A higher dosage of supports in the first year increased the odds of adequate implementation fidelity; therefore, program developers and implementation agents should ensure that in the early phases of implementation, there is a sufficient dosage of support provided to school teams.

## Supplementary Information

Below is the link to the electronic supplementary material.

Supplementary file1 (DOCX 25 KB)

## Data Availability

De-identified data, codebooks, and data collection instruments will be available at https://www.icpsr.umich.edu/web/pages/NACJD/index.html.
